# 

**Published:** 1896-09-19

**Authors:** 


**CADBURY'S COCOA**
"The standard of highest purity at presen attainable."—*Lancet.*

**CADBURY'S COCOA**

NO ALKALIES USED.

### THE HOSPITAL

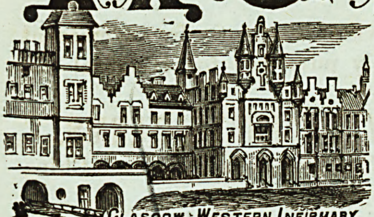

GLASGOW WESTERN INFIRMARY

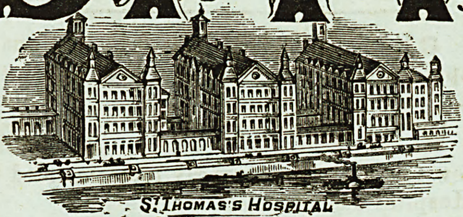

ST THOMAS'S HOSPITAL

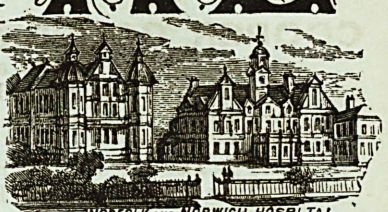

NORFOLK AND NORWICH HOSPITAL

No. 521. Vol. XX.

SATURDAY, SEPT. 19TH, 1896.

WITH EXTRA NURSING SUPPLEMENT.
[Registered at the General Post Office as a Newspaper for transmission in the United Kingdom and Abroad.]

